# Human-Pathogenic *Enterocytozoon bieneusi* in Captive Giant Pandas (*Ailuropoda melanoleuca*) in China

**DOI:** 10.1038/s41598-018-25096-2

**Published:** 2018-04-26

**Authors:** Wei Li, Zhijun Zhong, Yuan Song, Chao Gong, Lei Deng, Yuying Cao, Ziyao Zhou, Xuefeng Cao, Yinan Tian, Haozhou Li, Fan Feng, Yue Zhang, Chengdong Wang, Caiwu Li, Haidi Yang, Xiangming Huang, Hualin Fu, Yi Geng, Zhihua Ren, Kongju Wu, Guangneng Peng

**Affiliations:** 1 The Key Laboratory of Animal Disease and Human Health of Sichuan Province, College of Veterinary Medicine, Sichuan Agricultural University, Wenjiang, 611100 China; 2 Wolong Giant Panda Base, Aba, 624000 China; 3 Sichuan Key Laboratory of Conservation Biology for Endangered Wildlife, Chengdu Research Base of Giant Panda Breeding, Chenghua, 610057 China

## Abstract

Human and animal infections of *Enterocytozoon bieneusi* (*E. bieneusi*) have consistently been reported worldwide, garnering public attention; however, the molecular epidemiology of *E. bieneusi* in the giant panda remains limited. We surveyed captive giant pandas in China for the presence of *E. bieneusi* by using PCR and sequence analysis of the ribosomal internal transcribed spacer (ITS) revealing a 34.5% positive rate, with seven known genotypes (SC02, EpbC, CHB1, SC01, D, F, and Peru 6) and five novel genotypes (SC04, SC05, SC06, SC07, and SC08) identified. We similarly analyzed water samples, and *E. bieneusi* was detected in two samples, with genotype SC02 identified. Phylogenetic analysis revealed that CHB1 did not cluster with any recognized group, while the remaining genotypes belonged to group 1. The predominance of zoonotic group 1 genotypes indicates a public health threat that giant pandas could spread *E. bieneusi* to humans. The identification of *E. bieneusi* in water samples suggests giant pandas could contribute to water contamination. Effective control measures are therefore needed to minimize the contamination of the water and prevent a human microsporidiosis outbreak.

## Introduction

Microsporidiosis is an emerging infectious disease caused by microsporidial parasites, including the most common and environmentally ubiquitous species, *Enterocytozoon bieneusi*, which is responsible for ~90% of all cases of human microsporidiosis^1^. The first case of human *E. bieneusi* infection was reported in 1985^2^, and increasing numbers have been reported worldwide ever since. In humans, *E. bieneusi* infection can cause persistent diarrhea, malabsorption, and wasting diathesis in immunocompromised patients, particularly those suffering from acquired immunodeficiency syndrome (AIDS), who can develop life-threatening chronic diarrhea. Immunocompetent patients can also suffer from self-limiting diarrhea for up to one month^3–5^. In addition to infecting humans, this parasite also has a wide host range, including domestic animals and wildlife. However, there remains no reliable effective treatment for the disease^6^. Owing to their significance and potential threat to public health, microsporidia are on the National Institute of Allergy and Infectious Diseases (NIAID) Priority Pathogens List and are considered Category B pathogens (https://www.niaid.nih.gov/research/emerging-infectious-diseases-pathogens).

While *E. bieneusi* isolates cannot be discriminated morphologically, the internal transcribed spacer (ITS) region of the rRNA gene of *E. bieneusi* has a high degree of diversity. Thus, it is commonly used in many studies for the detection and identification of *E. bieneusi* genotypes. To date, over 200 genotypes have been identified and classified into eight groups (groups 1–8) based on phylogenetic analysis. Among these, genotypes belonging to group 1 are considered zoonotic and pose a major threat to humans, while those in other groups are regarded as having little or no public health significance.

Giant pandas (*Ailuropoda melanoleuca*) are bears native to south central China on the International Union for Conservation of Nature (IUCN) Red List and are considered living fossils. Currently, the number of captive giant pandas is just over 370 according to the results of the fourth national giant panda survey in China. There are many factors that have led this animal to be endangered, such as its low reproductive rate, loss of habitats, and lack of food. The common occurrence of parasites in giant pandas also poses a threat to their survival^7^. Moreover, in China, human *E. bieneusi* infections have been reported in several areas, with infection rates ranging from 0.2% to 11.9%^1,6,8–10^, and the transmission route remains unknown. While the giant panda could be a source of human microsporidiosis, the role of the giant panda in the transmission of *E. bieneusi* has been poorly investigated. Thus, we examined the occurrence of *E. bieneusi* in giant pandas and their potential role in the zoonotic transmission of human microsporidiosis.

## Results and Discussion

**Figure 1:**
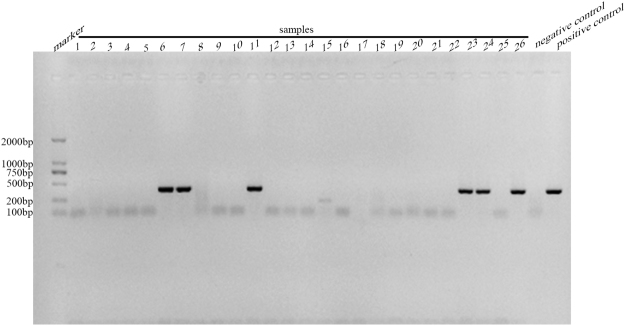
Amplification of partial samples in ITS locus. Full-length gels are presented in Supplementary Figure 1.

Of the 200 fecal samples collected from giant pandas, 69 were positive for *E. bieneusi*, with an overall infection rate of 34.5%. The result of agarose gel electrophoresis of PCR amplification products at ITS locus was shown in Fig. 1 (partial samples). The overall infection rate is almost four times higher than the rate among giant pandas at the Rare Wildlife Rescue Breeding Research Center and Xi’an Qinling Wildlife Park in Shaanxi province (8.7%)^11^. In addition, the infection rate observed in our study was higher than those reported in other animals in the order Carnivora in China, including 27.4% among Asiatic black bears (*Ursus thibetanus*), 13.89% among red pandas (*Ailurus fulgens*), 12.25–27.7% among foxes (*Vulpes lagopus*), 4.1–10.5% among raccoon dogs (*Nyctereutes procyonoides*), and 16.4% among blue foxes (*Alopex lagopus*)^11–15^. Our results therefore reveal the common occurrence of *E. bieneusi* infection in pandas. While pandas of different ages and genders were found to be infected with *E. bieneusi*, there were no significant differences in infection rates among yearling (20%), sub-adult (40%), and adult giant pandas (35.2%) (χ^2^ = 2.358, df = 2, P = 0.308) or between males (42.7%) and females (31.4%) (χ^2^ = 2.395, df = 1, P = 0.122) (Table 1). This is consistent with the results of a study conducted on *Cryptosporidium* in giant pandas, which also showed no significant differences in infections rates in terms of gender or age^16^.

**Table 1:** The occurrence of *E. bieneusi* in captive giant pandas in different age groups, genders, reservation bases and zoological gardens, China.

Category	No. samples collected	No. positive samples (%)	genotypes
Ages (year)			
<1.5 (yearling)	20	4(20%)	D(2),SC02(1),SC01(1)
1.5–5.5 (sub-adult)	35	14(40%)	SC02(10),SC06(2),SC04(1),F(1)
>5.5(adult)	145	51(35.2%)	SC02(39),EpbC(2),CHB1(2),SC01(1),SC04(1),SC07(1),SC05(1),SC08(1),D(1),F(1),Peru 6(1)
Gender			
female	105	33(31.4%)	SC02(24),SC04(2),EpbC(2),SC05(1),CHB1(1),SC01(1),SC06(1),D(1)
male	75	32(42.7%)	SC02(25),F(2),SC06(1),SC07(1),SC08(1),Peru6(1),CHB1(1)
unknown	20	4(20%)	SC02(1),SC01(1),D(2)
Reservation bases			
Chengdu research base of giant panda breeding	75	25(33.3%)	SC02(14),D(3),SC04(2),SC01(1),SC05(1),CHB1(2),F(1),EbpC(1)
Dujiangyan giant panda base	30	16(53.3%)	SC02(12),SC06(2),SC07(1),EbpC(1)
Wolong gengda giant panda base	37	7(18.9%)	SC02(5),SC08(1),F(1)
Yaan bifengxia giant panda base	17	4(23.5%)	SC02(3),SC01(1)
Wolong hetaoping giant panda base	10	3(30%)	SC02(3)
Zoological gardens			
Qingdao zoological gardens	2	1(50%)	SC02(1)
Shenzhen safari park	2	2(100%)	SC02(2)
Shanghai wild animal park	3	2(66.7%)	SC02(2)
Wenling changyu dongtian scenic spot	2	2(100%)	SC02(2)
Fuzhou giant panda zoo	4	2(50%)	SC02(2)
Ningbo zoological garden	2	2(100%)	SC02(2)
Changsha ecological zoo	2	2(100%)	SC02(2)
Chengdu zoological garden	3	1(33.3%)	Peru 6(1)
Liuzhou zoo	2	0(0)	
Wuxi zoological garden	1	0(0)	
Hefei wild animal park	1	0(0)	
Anji bamboo gardens	2	0(0)	
Hangzhou wild animal park	2	0(0)	
Nanjing hongshan forest zoo	2	0(0)	
Nanchang zoological garden	1	0(0)	
**Total**		69(34.5%)	SC02(50),D(3),SC06(2),CHB1(2),F(2),EbpC(2),SC01(2),SC04(2),SC05(1),SC07(1),SC08(1),Peru 6(1)

**Figure 2:**
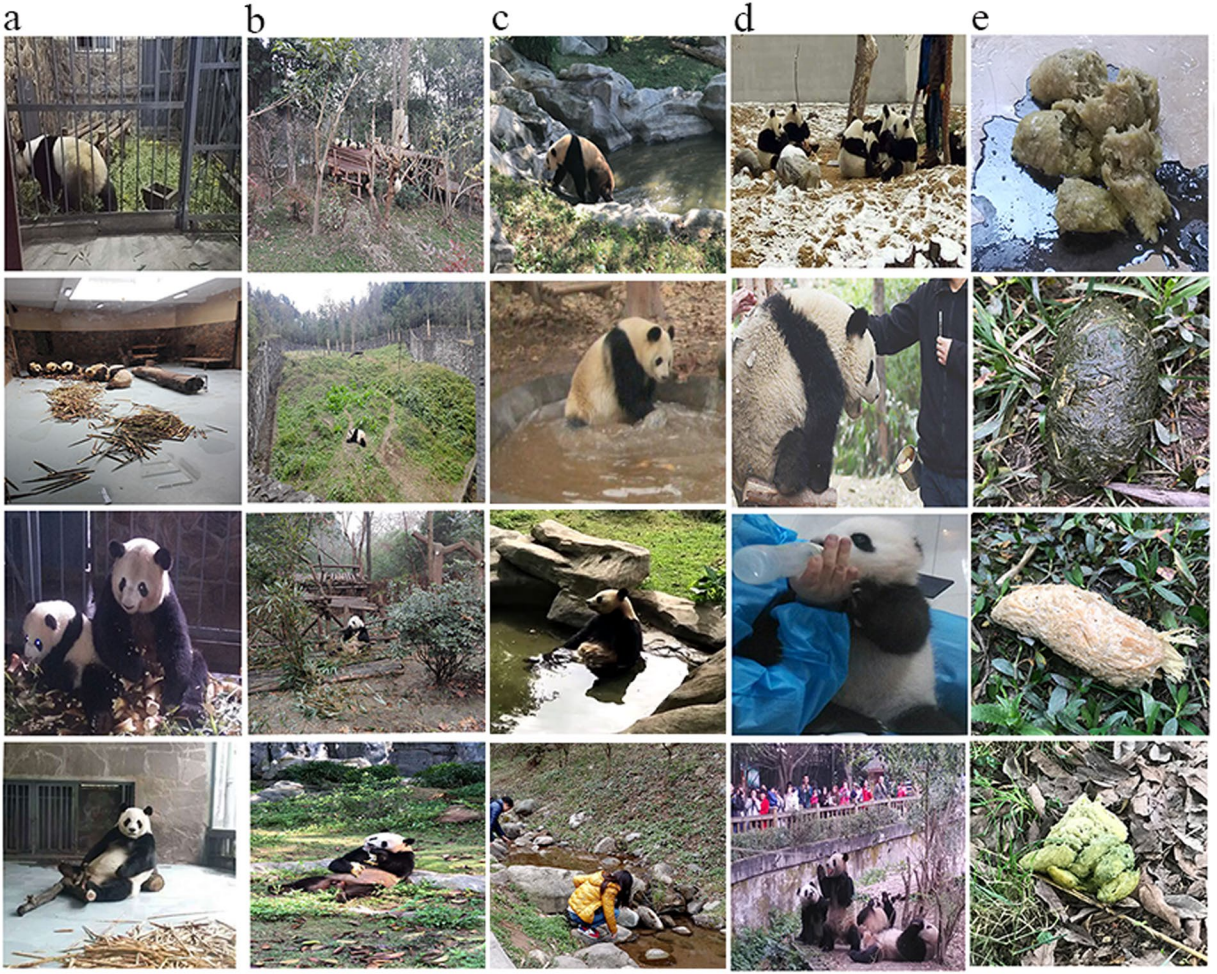
Images of living conditions of giant pandas and their feces, demonstrating their close contact with humans and water sources. (**a**) Typical indoor residence. (**b**) Typical outdoor residence. (**c**) Close contact between giant pandas and water or tourists and water (**d**) Examples of close contact between giant pandas and humans. (**e**) The feces of giant pandas.

In China, several conservation bases and zoological gardens have been built to protect endangered giant pandas. Here we collected 169 and 31 fecal samples from conservation bases and zoological gardens, respectively. An infection rate of 32.5% (55/169) was observed among conservation bases, while an infection rate of 45.2% (14/31) was observed among zoological gardens. No significant difference was found in terms of the infection rates of the conservation bases and zoological gardens (χ^2^ = 1.845, df = 1, P = 0.174) (Table 1). The data showed that *E. bieneusi* was found in 5/5 (100%) conservation bases and 8/15 (53.3%) zoological gardens (Table 1), with infection rates ranging from 0% to 100%. The highest infection rate (100%) was found in four zoological gardens, including Shenzhen safari park, Wenling changyu dongtian scenic spot, Ningbo zoological garden, and Changsha ecological zoo (Table 1). The typical indoor or outdoor residences for giant pandas differed among the sampled sites. Some indoor residences are spacious, and some giant pandas are often housed together for improved viewing by the public (Fig. 2a). In contrast, some of the indoor space is usually relatively narrow and pandas are typically housed separately; sometimes, bamboo nearly covers the entire floor, requiring the giant pandas to sit on the bamboo (Fig. 2a). Generally, the outdoor residences are surrounded by trees and planted with grasses or include the placement of large stones (Fig. 2b). These differences may contribute to the difference in observed infection rates, which may also be influenced by factors such as animal health status, sample size, and geographic location.

To the best of our knowledge, only two *E. bieneusi* genotypes have so far been identified in giant pandas: Peru 6 (n = 1) (found in Sichuan province) and type I-like genotype (n = 4) (found in Shaaxi province)^3,11^. In the present study, we identified seven known genotypes (SC02, EpbC, CHB1, SC01, D, F, and Peru 6) and five novel genotypes (SC04, SC05, SC06, SC07, and SC08), with SC02 (n = 50) being predominant, followed by genotypes D (n = 3), SC06 (n = 2), CHB1 (n = 2), F (n = 2), EbpC (n = 2), SC01 (n = 2), and SC04 (n = 2); SC05, SC07, SC08, and Peru 6 were only detected in one specimen each (Table 1). The wide identification of these genotypes in giant pandas in our study may due to that our *E. bieneusi* prevalence study was conducted on a larger scale with a broader survey area.

Among the seven known genotypes found in giant pandas, the most prevalent, SC02, has been previously reported in other animal hosts (Asiatic black bear, Tibetan blue bear (*Ursus arctos pruinosus*), Malayan sun bear (*Helarctos malayanus*), horse, and red-bellied tree squirrels (*Callosciurus erythraeu*)^3,12,17^, and it has also been found in humans (GenBank accession number KY465443). The giant panda belongs to the family Ursidae; in studies reporting infections of *E. bieneusi* in this family, several genotypes have been identified, including Peru 6, CHB1, SC02, horse2, ABB1, ABB2, and J. These results indicate that cross-species transmission may be occurring. Among the genotypes observed in humans in China, in addition to SC02 (GenBank accession number KY465443), genotypes EbpC and D have been described^1,6,8,9^. The presence of SC02 and D in giant panda may pose a threat to the public.

**Figure 3:**
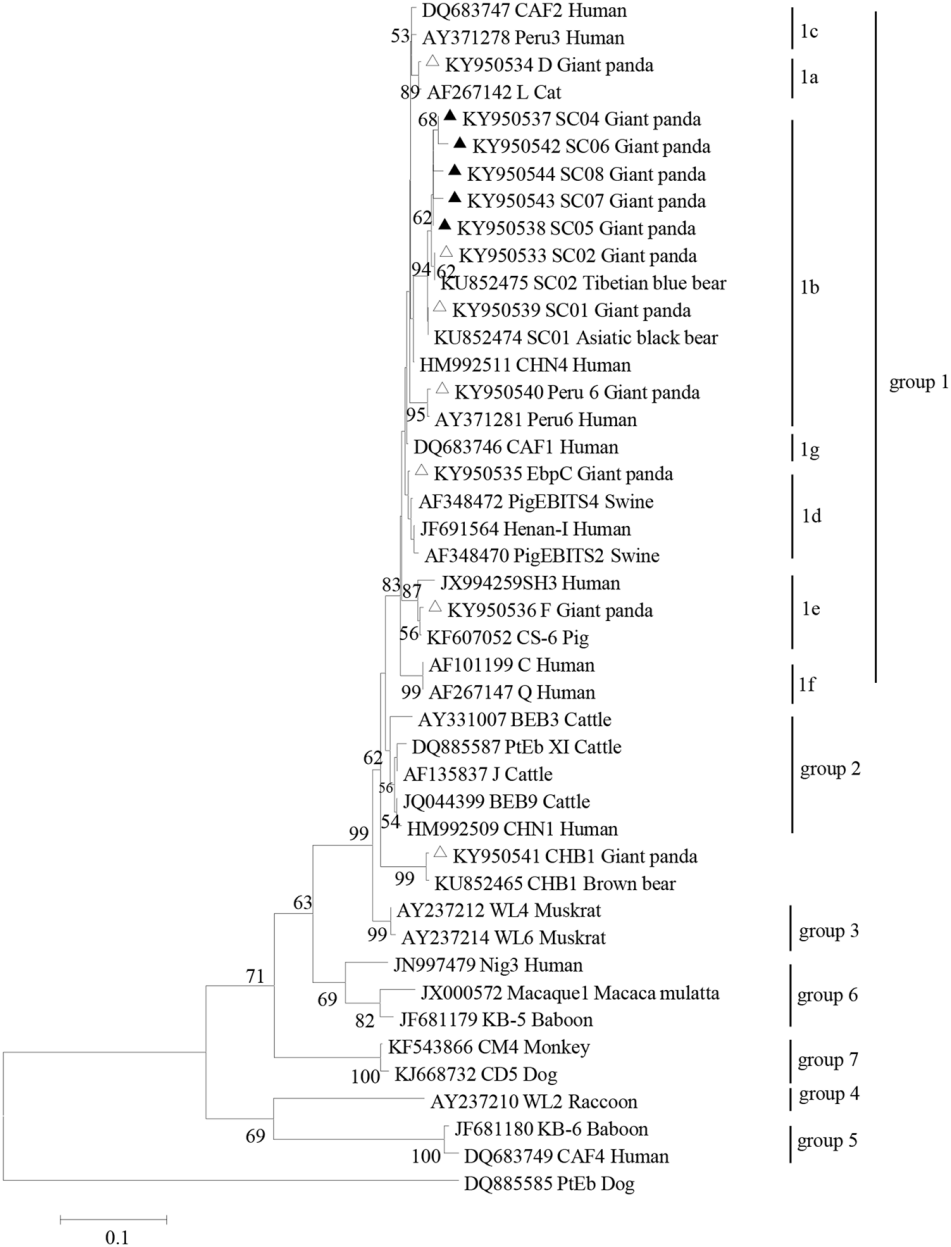
Phylogenetic relationships of ITS nucleotide sequences of the *Enterocytozoon bieneusi* genotypes identified in this study and other reported genotypes. The phylogeny was inferred by a neighbor-joining analysis. Bootstrap values were obtained using 1,000 pseudo-replicates and those greater than >50% were shown on nodes. The genotypes identified in this study are marked by triangles and the novel genotypes are marked by full triangles.

Phylogenetic analysis clustered all of the novel genotypes as well as SC01, SC02, and Peru 6 into group 1b. Genotypes D, EbpC, and F were distributed in groups 1a, 1d, and 1e, respectively. CHB1 did not cluster with any of the known E. bieneusi genotype groups (Fig. 3). With the exception of CHB1, all genotypes fell into zoonotic group 1, suggesting a potential threat to humans. None of the identified genotypes has been previously reported in giant pandas (except Peru 6), indicating that this represents a newly discovered host of these *E. bieneusi* genotypes.

Several countries have reported the identification of *E. bieneusi* in water samples, including Brazil, China, the USA, Ireland, and France^18–23^. In the present study, two water samples were positive for *E. bieneusi* collected in Chengdu research base of giant panda breeding located in Chengdu city. Previously, *E. bieneusi* have been detected in other cities, including Guiyang, Zhenzhou, Shanghai, Wuhan, Nanjing, and Qingdao, with over 40 genotypes detected^18,19,24,25^. In our study, only genotype SC02 was identified in water samples which was the dominant genotype found in giant pandas. Also the genotypes D, EbpC, and Peru 6 detected in our study (fecal samples) have been previously found in water samples from China^19,24,25^. The presence of SC02 genotype in water may support that giant pandas could be a source of water contamination due to the close contact between giant pandas and water (Fig. 2c).

The transmission of microsporidia can include airborne, person-to-person, zoonotic, and waterborne routes^26^. Our findings imply that giant pandas harboring *E. bieneusi* have the potential to spread this infection to humans and to contaminate water supplies. Individuals that maintain close contact with giant pandas, including feeders, veterinarians, and volunteers worldwide, may be susceptible to infection with *E. bieneusi* (Fig. 2d). Microsporidiosis is considered a serious human disease of waterborne origin, along with cryptosporidiosis and giardiasis^27^. Indirect zoonotic transmission of microsporidia between animals and humans could therefore occur through exposure to contaminated water. Nearly 90% documented outbreaks of these pathogens are associated with water^25^. Given the large number of visitors to conservation bases and zoological gardens and their exposure to potentially contaminated water (Fig. 2c,d), human infections could become endemic. Moreover, in China, the nutrition-rich feces of giant pandas are often used as fertilizer; thus, feces containing *E. bieneusi* spores could heavily contaminate the environment (soil and water), further contributing to the spread of *E. bieneusi*. Notably, as the giant panda is considered one of China’s national treasures, some giant pandas are sent to foreign countries, which may broaden the geographic range of *E. bieneusi* and facilitate the global transmission of this pathogen.

## Conclusions

Our study reveals the common occurrence of *E. bieneusi* infection (34.5%) in captive giant pandas in China and reports 11 *E. bieneusi* genotypes identified for the first time in giant pandas. Zoonotic group 1 genotypes predominated among giant panda isolates, indicating a potential public health threat. We also detected *E. bieneusi* in water samples genotyped as SC02. Our study indicates that giant pandas could spread *E. bieneusi* to humans and be a source of water contamination. Further research on the contamination of more water samples and the potential spread of *E. bieneusi* to humans is needed to elucidate *E. bieneusi* transmission routes.

## Methods

### Ethics approval and consent to participate

This study complied with the guidelines of the Regulations for the Administration of Affairs Concerning Experimental Animals and was approved by the Animal Ethical Committee of Sichuan Agricultural University. No animals were harmed during the sampling process. Permission was obtained from China Giant Panda Protection and Research Center for the collection of fecal specimens. All the procedures were carried out in accordance with the approved guidelines.

### Sample collection

From May 2016 to June 2017, 200 fecal samples were collected from captive giant pandas from five conservation bases (n = 169) and 15 zoological gardens (n = 31) in China. Eight water samples were collected from Chengdu research base of giant panda breeding where the fecal samples were mainly collected. Also, samples were collected from water ponds where giant pandas took bath. All samples were placed on ice in separate containers that were marked with sample information such as gender, age (for fecal samples) and collection location (for water samples) and then were transported to the laboratory immediately. None of giant pandas had any apparent diarrhea at the time of sampling (Fig. 2e).

### DNA extraction and nested PCR amplification

We used E.Z.N.A.® Tool DNA Kits (D4015-02; Omega Bio-Tek Inc., Norcross, GA, USA) for DNA extraction following the manufacturer’s protocol. For fecal samples, genomic DNA was extracted directly from approximately 200 mg of fecal sample. Whereas, for each water sample, prior to DNA extraction, we concentrated the pathogen by centrifugation at 6000 × g for 10 min as described previously^19^ and 300 μl sample concentrates were used for DNA extraction. The DNA samples were stored in 200 μl of the solution buffer from the kit at −20 °C until use.

The ITS gene was amplified for the identification of *E. bieneusi* using primers and amplification conditions described by Sulaiman *et al*.^28^. To amplify the ITS region of the rRNA gene, we use primers 5′-GATGGTCATAGGGATGAAGAGCTT-3′ and 5′-AATACAGGATCACTTGGATCCGT-3′ for the primary PCR, and 5′-AGGGATGAAGAGCTTCGGCTCTG-3′ and 5′-AATATCCCTAATACAGGATCACT-3′ for the secondary PCR. The annealing temperature is 55 °C for both primary and secondary PCR. Secondary PCR products were visualized by staining with Golden View following 1% agarose gel electrophoresis.

### Sequence and phylogenetic analysis

Amplicons of the expected size (392 bp) were sent to Invitrogen (Shanghai, China) for sequencing. A two-directional sequencing method was applied to ensure sequence accuracy. All nucleotide sequences obtained in this study were aligned with *E. bieneusi* reference sequences downloaded from the GenBank database using Blast (https://blast.ncbi.nlm.nih.gov/Blast.cgi) and ClustalX software (version 1.83; http://www.clustal.org/). Phylogenetic analysis was performed by constructing a neighbor-joining tree using Mega 6 software (http://www.megasoftware.net/) based on the evolutionary distances calculated using a Kimura 2-parameter model. The reliability of the tree was assessed using bootstrap analysis with 1,000 replicates.

### Statistical analysis

Differences between the tested conservation bases and zoological gardens in infection rates and the prevalences among different age groups and genders were compared using Chi-square tests (χ^2^) conducted with SPSS version 17.0 software (SPSS Inc., Chicago, IL, USA). A P-value < 0.05 was considered significant.

### GenBank accession numbers

Representative sequences identified in this study were deposited in the GenBank database (KY950533-KY950544).

### Data availability

All data generated or analysed during this study are included in this published article and its Supplementary Information files.

## Electronic supplementary material

Supplementary Figure 1

## References

[1] Yang J (2014). *Enterocytozoon bieneusi* genotypes in children in Northeast China and assessment of risk of zoonotic transmission. J Clin Microbiol..

[2] Desportes I (1985). Occurrence of a new microsporidan: *Enterocytozoon bieneusi* n.g., n. sp., in the enterocytes of a human patient with AIDS. J Protozool..

[3] Li W (2016). Multilocus genotypes and broad host-range of *Enterocytozoon bieneusi* in captive wildlife at zoological gardens in China. Parasit Vectors..

[4] Fiuza VR (2016). Zoonotic *Enterocytozoon bieneusi* genotypes found in brazilian sheep. Res Vet Sci..

[5] Didier ES (2005). Microsporidiosis: an emerging and opportunistic infection in humans and animals. Acta tropica..

[6] Wang L (2013). Zoonotic *Cryptosporidium* species and *Enterocytozoon bieneusi* genotypes in HIV-positive patients on antiretroviral therapy. J Clin Microbiol..

[7] Zhang JS (2008). Parasite threat to panda conservation. EcoHealth..

[8] Wang T (2017). First survey of *Cryptosporidium*, *Giardia* and *Enterocytozoon* in diarrhoeic children from Wuhan, China. Infect Genet Evol..

[9] Wang L (2013). Concurrent infections of *Giardia duodenalis*, *Enterocytozoon bieneusi*, and *Clostridium difficile* in children during a cryptosporidiosis outbreak in a pediatric hospital in China. PLoS Negl Trop Dis..

[10] Zhang X (2011). Identification and genotyping of *Enterocytozoon bieneusi* in China. J Clin Microbiol..

[11] Tian GR (2015). First report of *Enterocytozoon bieneusi* from giant pandas (*Ailuropoda melanoleuca*) and red pandas (*Ailurus fulgens*) in China. Infect Genet Evol..

[12] Deng L (2017). Multi-locus genotypes of *Enterocytozoon bieneusi* in captive Asiatic black bears in southwestern China: High genetic diversity, broad host range, and zoonotic potential. PLoS One..

[13] Zhang XX (2016). Prevalence, risk factors and multilocus genotyping of *Enterocytozoon bieneusi* in farmed foxes (Vulpes lagopus), Northern China. Parasit vectors..

[14] Zhao W (2015). Genotyping of *Enterocytozoon bieneusi* in farmed blue foxes (*Alopex lagopus*) and raccoon dogs (*Nyctereutes procyonoides*) in China. PLoS One..

[15] Yang Y (2015). Widespread presence of human-pathogenic *Enterocytozoon bieneusi* genotype D in farmed foxes *(Vulpes vulpes*) and raccoon dogs (*Nyctereutes procyonoides*) in China: first identification and zoonotic concern. Parasitol Res..

[16] Wang T (2015). Prevalence and molecular characterization of *Cryptosporidium* in giant panda (*Ailuropoda melanoleuca*) in Sichuan province, China. Parasit vectors..

[17] Li J (2015). Molecular Characterization of *Cryptosporidium spp*., *Giardia duodenalis*, and *Enterocytozoon bieneusi* in Captive Wildlife at Zhengzhou Zoo, China. J Eukaryot Microbiol..

[18] Ye, J., Ji, Y., Xu, J., Ma, K. & Yang, X. Zoonotic *Enterocytozoon bieneusi* in raw wastewater in Zhengzhou, China. *Folia parasitologica*. **64** (2017). 10.14411/fp.2017.002 28148905

[19] Li N (2012). Molecular surveillance of *Cryptosporidium spp*., *Giardia duodenalis*, and *Enterocytozoon bieneusi* by genotyping and subtyping parasites in wastewater. PLoS Negl Trop Dis..

[20] Yamashiro S (2017). Enterocytozoon bieneusi detected by molecular methods in raw sewage and treated effluent from a combined system in Brazil. Memorias do Instituto Oswaldo Cruz.

[21] Lucy FE, Graczyk TK, Tamang L, Miraflor A, Minchin D (2008). Biomonitoring of surface and coastal water for *Cryptosporidium*, *Giardia*, and human-virulent microsporidia using molluscan shellfish. Parasitol Res..

[22] Graczyk TK, Sunderland D, Tamang L, Lucy FE, Breysse PN (2007). Bather density and levels of *Cryptosporidium*, *Giardia*, and pathogenic microsporidian spores in recreational bathing water. Parasitol Res..

[23] Coupe S (2006). Detection of *Cryptosporidium*, *Giardia* and *Enterocytozoon bieneusi* in surface water, including recreational areas: a one-year prospective study. FEMS Immunol Med Microbiol..

[24] Ye J (2012). Anthroponotic enteric parasites in monkeys in public park, China. Emerg Infect Dis..

[25] Huang C (2017). Environmental transport of emerging human-pathogenic *Cryptosporidium* species and subtypes through combined sewer overflow and wastewater. Appl Environ Microbiol..

[26] Izquierdo F (2011). Detection of microsporidia in drinking water, wastewater and recreational rivers. Water Res..

[27] Graczyk TK, Majewska AC, Schwab KJ (2008). The role of birds in dissemination of human waterborne enteropathogens. Trends Parasitol..

[28] Sulaiman IM (2003). Molecular characterization of microsporidia indicates that wild mammals Harbor host-adapted *Enterocytozoon spp*. as well as human-pathogenic *Enterocytozoon bieneusi*. Appl Environ Microbiol..

