# Supplementary material for: Human-Pathogenic Enterocytozoon bieneusi in Captive Giant Pandas (Ailuropoda melanoleuca) in China
Source: Sci Rep. 2018 Apr 26;8:6590. doi: 10.1038/s41598-018-25096-2 (PMC5920105; doi:10.1038/s41598-018-25096-2)
Supplement: Supplementary file 1 — Supplementary Figure 1 [file 41598_2018_25096_MOESM1_ESM.pdf]

Human-Pathogenic *Enterocytozoon bieneusi* in Captive Giant Pandas (*Ailuropoda melanoleuca*) in China

Wei Li[1,+], Zhijun Zhong[1,+], Yuan Song[1,+], Chao Gong[1,+], Lei Deng[1], Yuying Cao[1], Ziyao Zhou[1], Xuefeng Cao[1], Yinan Tian[1], Haozhou Li[1], Fan Feng[1], Yue Zhang[1], Chengdong Wang[2], Caiwu Li[2], Haidi Yang[2], Xiangming Huang[3], Hualin Fu[1], Yi Geng[1], Zhihua Ren[1], Kongju Wu[3], Guangneng Peng[1*]

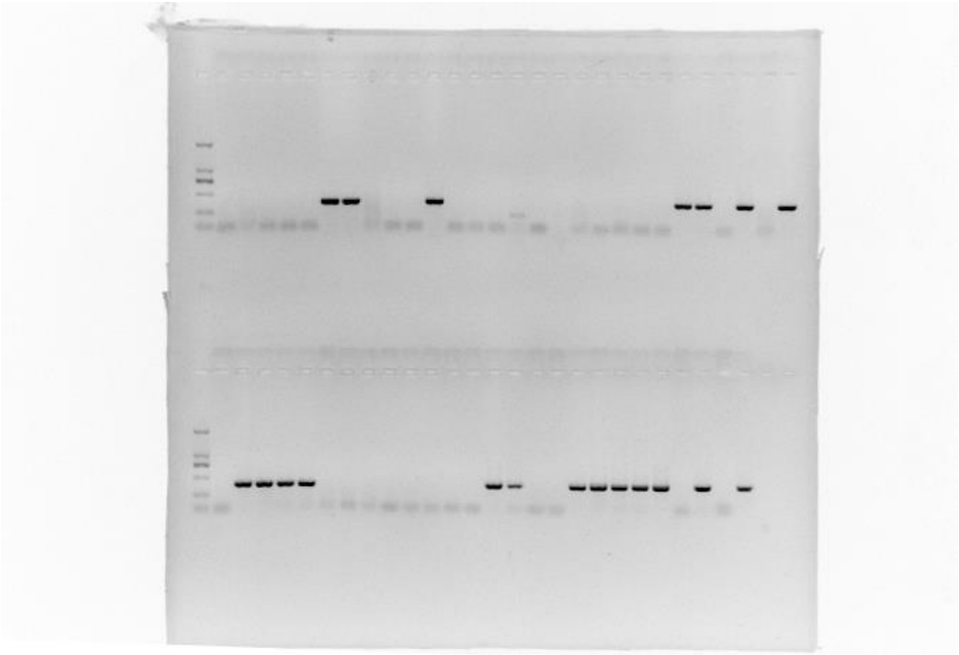

Supplementary Figure 1. Amplification of partial samples in ITS locus
